# The immunological bases of alemtuzumab as induction-therapy in pediatric-onset multiple sclerosis

**DOI:** 10.3389/fimmu.2024.1509987

**Published:** 2025-01-08

**Authors:** Marco Puthenparampil, Marta Gaggiola, Francesca Rinaldi, M. Nosadini, S. Sartori, Paola Perini, Paolo Gallo

**Affiliations:** ^1^ Department of Neurosciences, University of Padua, Padua, Italy; ^2^ Multiple Sclerosis Centre, Azienda Ospedaliera di Padova, Padua, Italy; ^3^ Immune-Mediated Nervous System Disease Study Group, Fondazione Istituto di Ricerca Pediatrica Città della Speranza, Padova, Italy; ^4^ Pediatric Neurology and Neurophysiology Unit, Department of Women’s and Children’s Health, University Hospital of Padova, Padua, Italy

**Keywords:** pediatric-onset MS, POMS, alemtuzumab, induction therapy, multiple sclerosis

## Abstract

Pediatric-Onset Multiple Sclerosis (POMS) is characterized by both white and grey matter inflammation, as well as by a higher risk of long-term physical and cognitive disability. The peculiar immunopathogenic mechanisms of POMS suggests that the use of induction therapies, including alemtuzumab (ALTZ), might be a promising approach, at least for postpuberal (> 11 yo) POMS. Although no data on the use of induction therapies in POMS are available from clinical trials currently, case series or case reports on the effect of alemtuzumab (ALTZ) have been recently published. In this review we have briefly revised the immunopathogenic features of POMS, as well as on how ALTZ might impact on them, reporting its efficacy observed in different POMS cohorts.

## Pediatric-onset multiple sclerosis: clinical aspects

Acquired demyelinating syndrome (ADS) in children under 18 years of age may represent various neuroinflammatory conditions. Among them, Multiple sclerosis (MS) is a chronic autoimmune, demyelinating, and neurodegenerative disease of the central nervous system (CNS). Pediatric-onset MS (POMS), defined when the disease presents clinically under the age of 18, identifies 3–5% of patients with MS, while less than 2% of POMS have an onset under 10 years of age (1, 2). Therefore, pediatric MS is a rare disease, much less common than adult-onset MS (AOMS).

Clinical onset of POMS is often characterized by optic neuritis, transverse myelitis, brainstem syndromes, or an acute disseminated encephalomyelitis (ADEM)-like event. Although many of the focal or multifocal neurologic presentations of POMS resemble those seen in AOMS, brainstem and cerebellar syndromes are particularly common in young children and adolescent patients (3, 4). Moreover, POMS are also much more likely to present with encephalopathy with fever, seizures, and/or polyfocal symptoms, thus mimicking ADEM (5). Although 50% of POMS patients enter the secondary progressive phase of MS after a median period of 23 years, i.e., a time 10 years longer than the observed in AOMS (2), POMS are likely to experience progressive disability at a younger age. In addition, cognitive sequelae of POMS can develop earlier during the disease course, are not associated to physical disability, and are characterized by an impairment in working memory, executive function, and processing speed (6–8).

The above summarized clinical characteristics of POMS define an unfavorable prognosis, that, however, can be influenced by the quality of treatment. Indeed, a reduction of 50-70% of persistent disability has been described in a study that recruited 3198 POMS followed for 21.8 ± 11.7 years (9). The risk of disability (i.e., reaching EDSS score ≥4.0 or ≥6.0) was associated with the disease duration at first EDSS evaluation, the male sex and the availability of new high efficacy therapies at the time of assessment (before 1993 *vs* 1993-1999 *vs* 2000-2006 *vs* 2007-2013) (9). These findings indicate the need of appropriate therapeutic algorithms to prevent disability and disease progression in POMS.

Beside MS, ADS in children under 18 years of age may also include anti-aquaporin-4-associated neuromyelitis optica spectrum disorder (AQP4-NMOSD), myelin oligodendrocyte glycoprotein antibody-associated disorder (MOGAD), or acute disseminated encephalomyelitis (ADEM) with encephalopathy (10). Although studies over the past decade have established that these are distinct entities, clinical phenotypic overlaps can occur between MOGAD, AQP4-NMOSD, and MS. However, cumulative biological, clinical, and pathological evidence allows for differentiation between these conditions (11). Notably, accurate diagnosis at the onset of ADS is crucial, as several studies have shown that baseline disease-modifying therapies (DMTs) for MS, such as interferon beta, glatiramer acetate, and natalizumab, are ineffective in preventing relapses in MOGAD and NMOSD (12). Therefore, to rule out these pathologies is mandatory and constitute the first step for choosing the best therapeutic option in children with MS.

## Different immunological mechanisms between pre- and post- puberal POMS

Although studies on the pathology of POMS are extremely rare, the available literature data highlight that prepuberal and post-puberal POMS have relevant pathological differences. Compared to AOMS, brain biopsy and autopsy samples from 19 children with POMS disclosed a more pronounced acute axonal damage in inflammatory demyelinating lesions, that was more pronounced in pre-pubertal (before 11 y.o.) than in post-puberal age (13). In both cases, axonal damage associated with macrophage rather lymphocyte (CD3-positive T cells or CD8-positive cytotoxic T cells) count, but the highest number of macrophages was measured in early active demyelinating lesions of pre-pubertal patients.

Consistent with these pathological findings, differences in clinical presentation, laboratory and imaging findings between prepubertal and pubertal MS onset were reported. Children with disease onset before puberty are more likely to present with a moderate to severe clinical pictures characterized by encephalopathy and/or multifocal symptoms (14–18). Furthermore, fever and impaired cognitive functioning are more common in younger children (14).

Cerebrospinal fluid (CSF) profile is also modified by age at disease onset (15): patients under 11 years of age have a higher percentage of polynuclear cells and monocytes, as well as a lower percentage of lymphocyte in the CSF. Furthermore, post-puberal POMS are more likely to show intrathecal synthesis of IgG oligoclonal bands or elevated IgG index than post-puberal POMS (19, 20). Abnormalities in T cell phenotype and function have been reported in AOMS (21), whereas relatively limited cellular immunology data are available in POMS. An early study suggested that children with early MS exhibited abnormally heightened circulating T cell responses to CNS autoantigens. A subsequent study assessed responses of T cells of both AOMS and POMS to Myelin Basic Protein (MBP) and Myelin Oligodendrocyte Glycoprotein (MOG) and found that both groups mounted preferential but similar responses to particular antigenic epitopes, including MBP_83–102_, MBP_139–153_, and MBP_146–162_ and MOG_1–26_, MOG_38–60_, and MOG_63–87_ (22). Intriguingly, T-cell was determined in peripheral blood mononuclear cells without any cell-sorting, suggesting a high frequency of self-reactive T-cell clones. POMS also exhibited higher frequencies of proliferating memory CD4+ T cells and higher levels of interleukin-17 secretion in response to myelin peptides than healthy children, suggesting that this T cell population may be relevant to pathogenesis of POMS (23). Taken all together, literature data indicate that POMS patients have an increased frequency of self-reactive T-cells in blood, independently of the puberal status. In addition, pre-puberal POMS patients have a higher activation of innate immunity. Therefore, induction therapies that target different immunopathological mechanisms have a rational in POMS. Since a shift in the incidence of MS after puberty, from a 1:1 ratio to a distinct female predominance, a pivotal role of sex hormones in the etiopathogenesis of the disease has been indicated (24).

## Therapeutic strategies for POMS

Although an increasing number of disease-modifying treatment options are available for patients with POMS, the above reported differences from AOMS in immunopathological, clinical, and radiological features question whether the efficacy and safety of DMT used to treat AOMS should be uncritically applied to POMS (25). Indeed, the efficacy of recently approved drugs (*fingolimod*, *teriflunomide*, *dimethyl fumarate*), that are considered treatment having a moderate efficacy in AOMS, was limited on the appearance of new/enlarging white matter lesions in POMS (i.e., annualized rate in Fingolimod-treated POMS 4.397; number per scan in Teriflunomide-treated POMS: 4.78; new/enlarging T2 lesion at week 96 in Dimethyl Fumarate-treated POMS: 12.49), thus calling for the use of more effective treatments.) (26–28). On the other hand, the high effect of natalizumab on clinical and radiological outcomes has been largely described, suggesting its use as first treatment in POMS in an escalation view of treatment (29, 30). Although the relevance of highly effective treatments in POMS have been already supported, clinical trials on ocrelizumab and ofatumumab are ongoing (a Phase 1, NCT02200718 and a Phase III Clinical trial, NCT05123703), while the experience on natalizumab is mainly derived from cohort studies (31–34).

Nevertheless, the peculiar immunological background of POMS might differentiate the effect of a drug between AOMS and POMS. This is particularly relevant for induction therapies (i.e., alemtuzumab (ALTZ), cladribine and autologous hematopoietic stem cell transplantation), whose mechanisms of action imply a marked and possibly long-lasting effect on adaptive immune system (i.e., B and T-cell receptor repertoire and network) (35).

## The effect of alemtuzumab on adaptive immune system

ALTZ is a humanized monoclonal antibody that targets CD52, a surface molecule mainly expressed by T-and B-cells (36), inducing the death of these cell by an antibody-dependent cellular cytotoxicity mechanism (37).

Administered in two courses of respectively 5 and 3 days distanced by 12 months, ALTZ determines an extensive depletion of lymphocytes, followed by a so called ‘reconstitution phase’ (38–41). The effect of ALTZ on the immune system persists in absence of further drug-exposure, determining long-term control on disease reactivation risk (42–44).

A great emphasis was initially given to the effect of ALTZ on the quantitative difference between B- and T-cell repopulation (45). Indeed B-cell repopulation is fast and determines a rapid, progressive increase of B-cell count, that returns to baseline values after 6 months and increases up to 20-30% from baseline values at month 12 (45, 46). On the other hand, T-cell reconstitution takes longer time. Indeed, CD4+ and CD8+ T-cells are still reduced 12 months after alemtuzumab infusion (-70% and -50% compared to baseline values respectively). T-Helper-1 and -17 are particularly reduced by ALTZ (47), but then T-cell count progressively increases partially for thymic output, mainly for homeostatic proliferation (48). T-reg subset reconstitution is faster than T-naïve and T-memory cells (49), leading to a hypothesized window (between month 6 and 12 after ALTZ infusion) during which the activation of survived (and potentially self-reactive) T-cells is slowed down (50). Interestingly, it was demonstrated that T-regs reconstitution is not driven by thymic output, but again by homeostatic proliferation of survived T-regs and by conversion from residual T-cells during the early post-treatment phase (49). Indeed, the more rapid increase of Tregs compared with conventional T-Helper cells determine a higher percentage of Treg on CD4+ T-cells, that progressively increase from month 6 to month 12. The effect of alemtuzumab on T-reg subset was also evaluated *in vitro*, where ALTZ-exposed T cells displayed functional regulatory characteristics (anergy to stimulation with allogeneic dendritic cells and ability to suppress the allogeneic response of autologous T cells both with cell–cell contact and Interleukin-2 consumption) (49).

Also, a rapid increase of B-regs, expressed as both absolute number and percentage, was observed and paralleled T-regs expansion (51). In addition, B cells from treated patients secreted higher levels of Interleukin-10 and Brain-derived neurotrophic factor and were able to inhibit the proliferation of autologous conventional T-Helper cells (52).

Nevertheless, the dynamic of B- and T-cell repopulation was not able to explain the clinical and radiological disease reactivation, as well as the risk of autoimmune adverse events, questioning whether the clinical relevance of the repopulation dynamic was based on “quality” rather than on “quantity” (53). Indeed, B-cell reconstitution is mainly driven by the high bioavailability of B-cell activating factor (BAFF) that occurs immediately after ALTZ administration and complete B-cell depletion (54). BAFF bioavailability increases B-cell survival in bone-marrow and spleen and determines a rapid and progressive differentiated repopulation of all B-cell subsets: transitional B-cells repopulate first, followed by naïve B-cells and then memory B-cells, paralleling a progressive decrease of BAFF serum concentration (54). This repopulation mechanism explains why after ALTZ the variability of B-cell receptor repertoire is significantly increased. On the other hand, since T-cells repopulation is mainly driven by homeostatic proliferation of survived T-cells, T-cell Receptor repertoire is narrowed after ALTZ administration, and this effect is particularly relevant to predict the autoimmune adverse events (55–57), but might also explain the long-term effect on MS (48, 56).

## Efficacy and safety of alemtuzumab in POMS

The major limitation of effect of ALTZ in pre-pubertal POMS consists in the absence of any activity on natural immunity cells that do not express CD52. Moreover, no data are available in the literature on pre-pubertal POMS, but a clinical trial (NCT03368664) is ongoing recruiting patients between 10 and 18 y.o.

In a case series with two post-puberal patients, the authors report a stable EDSS after a short follow-up (37 month, 20 months, i.e. 24 months and 8 months after ALTZ last infusion), in absence of any relevant systemic autoimmune adverse event, radiological worsening or clinical relapse (58). In both cases patients were initially treated with teriflunomide 14 mg and then switched to ALTZ within the first year of treatment. Notably, six months after starting ALTZ, one patient developed a syndrome of presumed viral origin, characterized by fatigue and mild headache, in the absence of fever or signs of meningeal involvement, with complete recovery after two weeks (58).

In a different cohort, 2 POMS started ALTZ following natalizumab. One patient (patient 2 of the case series, disease onset: 9 y.o.) had three relapses in the 18 months on natalizumab and then switched to ALTZ (59). Four months later he had a severe relapse (EDSS 5.0), with complete recovery in 2 weeks. After the second cycle he was clinically and radiologically stable (follow-up: 7 months). The second patient (patient 5 of the case series, disease onset: 16 y.o.) was treated with fingolimod and rapidly switched to natalizumab for radiological activity. He developed two relapses and 4 new white matter lesions during the first 7 months of natalizumab, and thus switched to ALTZ (no data on anti-natalizumab antibody is reported). After the first ALTZ cycle, he had no evidence of clinical or radiological disease activity for 32 months.

Since treatments before ALTZ might influence its efficacy we have recently evaluated the efficacy and safety of ALTZ in a cohort of POMS that discontinued NTZ (60). Survival analysis revealed that only 1 patient (10% of the whole cohort) developed a clinical relapse 12 months after last ALTZ infusion, while 4 patients (40%) developed asymptomatic radiological disease activity during the follow up. No serious adverse event was observed. Considering the high disease activity rate before NTZ, the administration of ALTZ determined 36 months of No Evidence of Disease Activity 3 (NEDA-3) condition in 37.5% of patients, describing a good profile of efficacy and safety of ALTZ in post-puberal POMS. These data suggest that in POMS a maintenance therapy should be administrated earlier than in adults. On the other hand, the efficacy of ALTZ after NTZ was higher in AOMS. Indeed, only 2 patients developed clinical relapse during the follow up (one had the diagnosis of MS when she was 19 y.o.). Moreover, a higher percentage of AOMS (85.7%) achieved NEDA-3 at month 36 compared to POMS (p=0.05) (**Table 1**). In addition, the qualitative effect on the immunopathogenic mechanisms could be also hypothesized since the disease reactivation were mainly radiological, in line with the high impact on inflammatory parameters already observed in AOMS (61–63).

**Table 1:** Demographic variables of POMS treated with Alemtuzumab.

Ref.	Age at onset	Gender	Disease duration at ALTZ (y)	number of treatments before ALTZ	Previous treatment(s)	ALTZ course	Follow up (months)	Relapse after ALTZ
^57^	14	Male	1.7	1	Teri	1	2	no
^57^	8	Male	8.8	1	Teri	2	18	no
^58^	9	Male	5.5	1	NTZ	2	24	yes
^58^	16	Male	3.6	2	Fing, NTZ	2	32	no
^59^	12	Male	6.3	2	IFN, NTZ	2	44	yes
^59^	11	Female	6.9	1	NTZ	2	27	yes
^59^	12	Female	5.9	2	IFN, NTZ	2	54	yes
^59^	15	Female	3.5	1	NTZ	2	28	no
^59^	16	Female	2.8	1	NTZ	2	61	no
^59^	17	Male	8.1	1	NTZ	2	56	no
^59^	17	Male	2.0	1	NTZ	2	37	no
^59^	16	Male	3.7	2	IFN, NTZ	2	73	yes
^59^	17	Male	2.3	1	NTZ	2	74	yes
^59^	18	Female	1.7	1	NTZ	2	54	no

ALTZ, Alemtuzumab; Teri, Teriflunomide; NTZ, Natalizumab; Fing, Fingolimod; IFN, Interferon.

The different efficacy of ALTZ after NTZ in POMS might be explained by the high percentage of circulating self-reactive lymphocytes in POMS, which might limit the impact of induction therapies in these patients. Moreover, in post-NTZ POMS auto-proliferation may cause a significant expansion of the self-reactive repertoire, increasing the probability of survival of self-reactive T-cell after ALTZ. Interestingly, a more rapid reconstitution of the T cell repertoire is also observed in children compared to adults after autologous haemopoietic stem cell (64). Taken all together, these considerations suggest a more rapid homeostatic proliferation of survived T-cells (including self-reactive T-cell whose percentage is increased also by auto-proliferation) in POMS after ALTZ.

Piecing together the 14 POMS treated with alemtuzumab, we can observe that the risk of disease reactivation in higher in POMS treated with more than one drug before ALTZ (80%) compared with those who had ALTZ as second treatments (11.1%, Odds Ratio 32.00 95%IC 1.294 - 421.3, p=0.023).

## Future prospective

Post-puberal POMS might be eligible for ALTZ treatment, but clinical trials will define whether ALTZ might be equally effective in both pre- and post-puberal POMS. Specific condition (e.g, previous treatment with NTZ) might expand self-reactive repertoire, reducing the probability of eliminating all autoreactive lymphocytes. Biomarkers, such as self TCR/BCR expansion, are warranted to optimize treatment response, especially in POMS, tailoring personalized therapy in POMS.

## Conclusions

The high inflammatory activity that characterizes POMS requires the administration of highly efficacy treatments as soon as possible. While the use in ALTZ following NTZ should be planned with caution, its use in naïve post-pubertal POMS may have a relevant clinical impact. More data from RCT are needed in order to set more effective and safe therapeutic protocols.
